# 

**Published:** 1869-12

**Authors:** 


CONTENTS:

TRANSLATIONS:
Clinical Lectures upon Mental and Nervous Diseases, continued from Nov. number - - - - - 685
FOREIGN ITEMS:
Vaccinal Syphilis, - - - - 696
Ovariotomy in a Girl 12 years old, - 697
FOREIGN CORRESPONDENCE:
Letter from Dr. Bosworth, of Paris, - 701
ORIGINAL COMMUNICATIONS:
ART. I. Remarkable case of Monstrosity, with cut, - - 704
" II. Transfusion of Blood, Report of a Vivisection, - - 705
" III. Congenital Fibro-Cellular Tumor—By Moses Gunn, M.D., with cut, - - - 707
" IV. Alarming Dental Hæmorrhage. By N. H. Church, M.D., - - - - - 708
" V. Reversio. By J. E. O'B., M.D. 708
" VI. Sub-involution, wiih Retroflexion. By F. B Norcom, M. D. - - - - -
" VII. Corporeal Metritis, with Abscess of Broad Ligament. By F. B. Norcom, M. D. 712
" VIII. Cervical and Corporeal Endo-metritis, with Retroflexion By F. B. Norcom, M.D., - 714
ART. IX. A Case of Placenta Prævia. By J. P. Hamilton, M.D. 717
" X. Memoranda of an Obstetrical Case. By J. W. Brooks, M.D., - - - - - 718
" XI. Poisoning by Opium. By J. O'Brien, M.D., Pillipsburg, N. J., - - - - - 720
HOSPITAL REPORTS:
St. Luke's Hospital, - - - - - 721
EDITOR'S BOOK TABLE, - - - - 723
LITERARY AND SCIENTIFIC, - - - 731
MATERIA MEDICA, - - - - - 733
EDITORIAL:
The State Insane Hospital, - - 736
Women as Medical Students - - 737
Writing Prescriptions, - - - 738
LOOT:
Malarial Hæmaturia, - - - 740
Embolism of the Heart, &c., - - 742
Aqua Chlorini in Trichinitis - - 743
Deodorized Carbolic Acid, - - 743
Treatment of Delirium Tremens, - 743
Detection of Blood Stains, - - 744
Recipe for Burns, - - - - 745
Case of Dr. Schoeppe, - - - 745
Medical Record, - - - - 748
Milk, - - - - - - - - 748
MEDICAL JOURNAL ADVERTISER, - -

